# Uptake of core outcome sets by clinical trialists publishing in major medical journals: Protocol

**DOI:** 10.12688/hrbopenres.13109.2

**Published:** 2021-01-25

**Authors:** Karen Matvienko-Sikar, Kerry Avery, Jane Blazeby, Karen Hughes, Pamela Jacobsen, Jamie Kirkham, Jan Kottner, Katie Mellor, Ian Saldanha, Valerie Smith, Caroline B. Terwee, Paula R. Williamson, MRC-NIHR TMRP Outcomes Working Group Core Outcome Set Theme

**Affiliations:** 1 School of Public Health, University College Cork, Cork, Ireland; 2 NIHR Biomedical Research Centre, Bristol Medical School: Population Health Sciences, University of Bristol, Bristol, UK; 3 MRC/NIHR Trials Methodology Research Partnership, Department of Biostatistics, University of Liverpool, Liverpool, UK; 4 Department of Psychology, University of Bath, Bath, UK; 5 Centre for Biostatistics, Manchester Academic Health Science Centre,, University of Manchester, Manchester, UK; 6 Department of Dermatology and Allergy, Clinical Research Centre for Hair and Skin Science, Berlin, Germany; 7 Centre for Statistics in Medicine, University of Oxford, Oxford, UK; 8 Center for Evidence Synthesis in Health; Department of Health Services, Policy, and Practice, Brown University School of Public Health, Providence, Rhode Island, USA; 9 School of Nursing and Midwifery, Trinity College Dublin, Dublin, Ireland; 10 Amsterdam UMC, Department of Epidemiology and Biostatistics, Amsterdam Public Health Research Institute, Vrije Universiteit Amsterdam, Amsterdam, The Netherlands

**Keywords:** Core outcome sets, trials, uptake

## Abstract

**Background: **Outcome heterogeneity, selective reporting, and choosing outcomes that do not reflect needs and priorities of stakeholders, limit the examination of health intervention effects, particularly in late phase trials. Core outcome sets (COS) are a proposed solution to these issues. A COS is an agreed-upon, standardised set of outcomes that should be measured and reported as a minimum in all trials in a specific area of health or healthcare. COS are intended to increase standardisation of outcome measurement and reporting to better enable comparisons between, and synthesis of findings of trials in a particular health area.

**Methods: **This study will examine late phase trials, published between October 2019 and March 2020 (inclusive), in the following five medical journals: *New England Journal of Medicine*, *Journal of the American Medical Association*, *Lancet*, *BMJ*, and *Annals of Internal Medicine*. Trials will be examined to determine if they refer to a COS, and whether they use a COS. Trialists for each identified trial will subsequently be contacted to complete an online survey examining trialists’ awareness of, and decisions to search for and use a COS.

**Discussion: **This study will provide important information on uptake of COS by later phase trialists in major medical journals, and the views of these trialists on COS use in trials. These findings will inform approaches to increasing awareness and uptake of COS in future health trials.

## Introduction

Core outcome sets (COS) are standardised sets of agreed upon outcomes that should be measured and reported in all trials of a particular health area ^1^. COS represent the minimum outcomes that should be measured and reported to facilitate standardisation and to improve the examination of intervention effectiveness ^1, 2^. Heterogeneity in outcomes across trials has been noted as a significant problem in terms of evidence syntheses because not all outcomes can be compared across trials ^3^. Selective outcome reporting, or outcome reporting bias, which relates to inclusion of a subset of originally measured outcomes in the final publication is also problematic as it reduces research validity of trials and contributes to outcome heterogeneity ^4^. Outcome heterogeneity and selective reporting limit transparent examinations of intervention effects and contribute to research waste ^5^. In addition, outcomes included in trials do not always reflect those outcomes that are of importance to patients and other key stakeholders, such as healthcare professionals and policy makers ^6, 7^. COS represent an approach to minimising these problems by providing a minimum outcome set, that has been agreed by consensus by key stakeholders ^1^.

Development and use of COS is supported by the COMET Initiative, and resources including a COS handbook ^1^ and development and reporting guidelines ^8– 10^ are available. The development and use of COS in trials are increasing over time; the most recent update to a systematic review of COS development reported that over 300 COS studies have been published up to 2019, and at least 200 are currently being developed ^11^. Reviews of COS uptake indicate varying, though typically low, rates of COS use in trials ^12^. Use of COS to inform outcome choice in systematic reviews has been reported as just 7% in a recent 2020 review of 100 Cochrane reviews (Williamson *et al.* under review). Similarly, a recent examination of primary research applications to the National Institute for Health Research Health Technology Assessment (NIHR HTA) reported that 19% of applicants referenced a COS in relation to outcome choice ^12^. In this study, applicants reported that patient and public opinion, outcomes used in other studies, and recommendations from funders and/or professional bodies influenced outcome choice in funding applications ^12^. Though research has been conducted on uptake of individual COS, and COS in specific health areas ^13^, data on the use of COS in a general unselected cohort of published trials is lacking. Examinations of trialists’ views and perceived barriers and facilitators to using COS in trials are similarly lacking. This information is of importance to inform strategies to increase awareness and implementation of COS.

The aims of this study are to examine: (1) current practices of later phase trials published in top medical journals, in relation to the use of COS in choosing trial outcomes; and (2) views of trial authors on the use of COS in relation to choosing trial outcomes.

## Methods

### Search strategy

We will examine late phase trials published in the following journals: *New England Journal of Medicine*, *Journal of the American Medical Association (JAMA)*, *Lancet*, *BMJ*, and *Annals of Internal Medicine*. Each journal website will be searched across a 6-month period, from October 2019 to March 2020. Based on initial scoping of the target journals it is estimated that 115 trials, of various phases, have been published in these journals during this timeframe and so this period has been chosen to ensure identification of a sufficient, yet pragmatically manageable number of recent trials for review by the review team. In addition, this time frame ensures a sample of pre-COVID-19 trials (COVID-19 trials are being examined in a separate project in collaboration with https://covid-evidence.org).

### Inclusion/exclusion criteria

Late phase trials will be eligible for inclusion. For this study, late phase trials are defined as studies examining effectiveness of an intervention (pharmacological or otherwise), typically in relation to standard care or another comparator. In pharmacologic trials, these are typically referred to as phase III or phase IV clinical trials, though we are cognisant that this classification is not typically used in non-drug trials. In this study, late phase trials can include various trial designs (e.g. parallel, crossover, factorial, adaptive, and stepped-wedge designs) and with any level of randomisation (e.g. individual and cluster levels). There are no restrictions based on sample size, topic/health area, or intervention type. Trials will not be included if they are: feasibility trials aiming to examine whether some aspect of the trial or intervention can be done ^14^; pilot and exploratory trials preparing the conduct of the future trial, or part of the future trial, on a smaller scale ^14^; follow-up studies; or secondary analyses of late phase trials, or phase 1 and phase 2 studies. Systematic reviews and other study designs will also be excluded.

### Screening

Two reviewers (KMS & VS) will independently screen all identified trial publications to determine whether they are late phase trials meeting eligibility criteria for inclusion in the review. Discrepancies between reviewers will be resolved by consensus discussion or by recourse to a third reviewer (PW).

### Data extraction and analysis

A standardised data extraction form will be used for all articles, with data extracted by one reviewer and verified by a second reviewer. Extracted data will include: author, date, title, funding information including location of funder, study aims, disease or health category (using the COMET categories), disease name, target population, type of intervention used. Data will be extracted from the trial protocol or trial registry on whether a COS was mentioned. Data will also be extracted on whether a COS was mentioned and the reason for which it was mentioned (e.g. mentioned because it was used in trial, or mentioned to support a discussion point); whether a COS was used and if so, whether the full COS was used or whether only some COS outcomes were used. Details of the COS used will be extracted, including the individual outcomes used in trial that do not use the full COS. Whether the primary outcome of the trial was a COS, and if so which one, will also be extracted. For trials not reporting use of a COS, the trial authors’ rationale and justification for the choice of outcomes used will be extracted from the published trial if reported.

In addition, for trials not reporting COS use, we will examine whether a COS existed that could have been used at the time of trial commencement to determine trial outcomes. This will be done by first searching for a published protocol or trial registry entry (e.g. in ClinicalTrials.gov, ISRCTN registry) to identify an indication of when the trial started. As trials may begin prior to protocol publication/registration, this will be taken into account by extracting information on the start of trial recruitment from either the registry entry or the trial publication. The COMET database will then be searched by disease and health categories to identify whether a potentially relevant COS could have been used for each trial based on the timing of trial commencement and the timing of COS publication. Where a published protocol or trial registry entry cannot be identified to establish when the trial was being designed, the COMET database will be searched for a COS of relevant scope that had been published by 2017, such that it could potentially have informed choice of outcomes for the trial. We will check this assumption with the trialists (see below). A COS will be considered to be of relevant scope if it was developed for the same population (or a broader subset within which the trial population sits) and/or for the same intervention type (or a broader subset within which the trial intervention sits). Checking the COMET database will be done by one reviewer with prior experience of identifying COS for specific populations and interventions, and will be verified by a second reviewer.

### Survey of trialists

A survey will be sent to all corresponding authors of identified trials. When senior/corresponding authors cannot be contacted via emails, another author from the author list (i.e. first or last author) will be approached. The survey will examine trialists’ awareness of, and decisions to search for and use, a COS. An email will be sent to all trialists, including a link to the online survey, hosted on Google Forms® (see extended data ^15^). One of four versions of the survey will be sent as follows: 1) where trial publications mentioned a COS and the full COS was used; 2) where trial publications mentioned a COS and some but not all COS outcomes were used; 3) where trial publications do no mention a COS and we identified a potentially relevant COS that could have been used; and 4) where trial publications do no mention a COS and we did not identify a potentially relevant COS that could have been used. The surveys will ask about trialists’ identification and use of a COS, or not; experiences and issues with COS use where a COS was used; and reasons for choice of outcomes where a COS was not used.

### Analysis

Data collected from review of identified eligible trials and the survey of trialists will be analysed and presented descriptively. The main outcomes of this study will be the numbers and percentage of trials using a COS and the numbers and percentage of trials that could have used a COS. Secondary outcomes are trialists’ awareness of, and decisions to search for and use, a COS. Open-ended survey questions will be analysed used content analysis. Findings will be presented narratively and in tabular format.

### Ethical considerations and consent

Ethical approval is not necessary for examination of the published trials but is required for, and will be obtained prior to commencement of, the trialist survey. All participants will receive an electronic information leaflet and, following reading this, will provide electronic consent prior to completing the online survey. While it is not anticipated that the survey will cause any distress, the researchers contact details will be provided at the end of the survey should participants wish to discuss any issues raised or be provided with further support contact details.

### Dissemination

The findings of this study will be disseminated through the publication of peer-reviewed manuscripts. Additionally, findings will be presented at both national and international conferences.

### Study status

This study has not yet commenced.

## Discussion

Use of COS in trials is important to improve standardisation of outcomes, reduce bias and research waste, and improve examination and understanding of the effects of interventions in particular health areas. This study will provide information on the proportion of trialists in major medical journals who currently are, or are not, using COS. These findings will provide important insight into current uptake of COS in trials published in major medical journals. In addition, the study will provide information on trialists’ views and reasons for using, or not using, COS in trials. This is essential to better understand barriers and facilitators to COS uptake in medical trials.

## Data availability

### Underlying data

No data are associated with this article

### Extended data

Open Science Framework: Uptake of core outcome sets by clinical trialists publishing in major medical journals. https://doi.org/10.17605/OSF.IO/H4EKV ^15^

- COS TMRP Survey.pdf (Four versions of survey to be used in study)
- Uptake of COS by clinical trialists publishing in major medical journals.pdf (full study protocol document)

Data are available under the terms of the Creative Commons Attribution 4.0 International license (CC-BY 4.0).
