# Supplementary material for: Uptake of core outcome sets by clinical trialists publishing in major medical journals: Protocol
Source: HRB Open Res. 2021 Jan 25;3:53. Originally published 2020 Aug 10. [Version 2] doi: 10.12688/hrbopenres.13109.2 (PMC7919602; doi:10.12688/hrbopenres.13109.2)
Supplement: Peer review history

# Peer review: Reviewer response for version 2

**DOI:** 10.21956/hrbopenres.14343.r28748

**Published:** 2021-02-26

**Authors:** Christine Bond

**Affiliations:** 1 Institute of Applied Health Sciences, School of Medicine, Medical Sciences & Nutrition, University of Aberdeen, Aberdeen, UK

The authors have responded thoroughly and satisfactorily to all my comments. However their response does not always say if they also amended the actual paper. It looks as though my first comment on what this adds to the Cochrane review has not been explicitly added although it is implied. Similarly I don’t think anything has been added to take account of my second and third comments. Other than that all my comments seem to have been addressed by changes to the paper.

Is the study design appropriate for the research question?

Yes

Is the rationale for, and objectives of, the study clearly described?

Partly

Are sufficient details of the methods provided to allow replication by others?

Yes

Are the datasets clearly presented in a useable and accessible format?

Not applicable

Reviewer Expertise:

Mixed methods health services researcher who has been part of a team developing a COS.

I confirm that I have read this submission and believe that I have an appropriate level of expertise to confirm that it is of an acceptable scientific standard.

# Peer review: Reviewer response for version 1

**DOI:** 10.21956/hrbopenres.14215.r28034

**Published:** 2020-10-21

**Authors:** Anne Spinewine

**Affiliations:** 1 Louvain Drug Research Institute, Clinical Pharmacy Research Group, Université catholique de Louvain, Brussels, Belgium; 2 Pharmacy Department, CHU UCL Namur, Namur, Belgium

This is an interesting and well written protocol for a study evaluating the uptake of COS and exploring reasons for (not) using COS.

 The rationale is clearly justified, the methodological approach is sound and clearly written, the discussion is adequate.

 A few points for consideration are:

- The choice of the journals selected could be better described.
- Inclusion/exclusion criteria: would you also include trials with slightly different designs such as: stepped-wedge trials; adaptive trials?
- I suppose that SRs of trials would be excluded – although it would also be interesting to look if the use of COS is also addressed. Exclusion could be specifically mentioned.
- Data extraction: if the use of a COS is not mentioned in the main paper, would it be safe to check the detailed protocol (in some areas, it might have been previously published in another journal) if no reference is made to an existing COS.
- The search of existing COS is made in the COMET database. Is there any data to confirm that this is sufficient, i.e. that searching in parallel another database (eg PubMed) for published COS is not needed?
- Questionnaire: how will you maximize response rate? Has the questionnaire been pilot-tested? Will there be close and open-ended questions?
- The trialists’ views is essential to better understand the barriers and facilitators. Could there be any value of looking at some of the data extracted in the first part of the study, and identify from there factors associated with the (non)use of a COS? E.g. domain, type of funding, patient and public involvement…?

Is the study design appropriate for the research question?

Yes

Is the rationale for, and objectives of, the study clearly described?

Yes

Are sufficient details of the methods provided to allow replication by others?

Yes

Are the datasets clearly presented in a useable and accessible format?

Not applicable

Reviewer Expertise:

Use of medicines in older people. I have previous experience in developing a COS.

I confirm that I have read this submission and believe that I have an appropriate level of expertise to confirm that it is of an acceptable scientific standard.

# Response

**Published:** 2020-12-09

**Authors:** Karen Matvienko-Sikar

**Affiliations:** University College Cork, Ireland

**Reviewer comment 1**. This is an interesting and well written protocol for a study evaluating the uptake of COS and exploring reasons for (not) using COS.

 The rationale is clearly justified, the methodological approach is sound and clearly written, the discussion is adequate.

 A few points for consideration are:

 The choice of the journals selected could be better described.

**Author response 1. **Thank you for your comments and useful feedback, which has now been incorporated.

**Reviewer comment 2. **Inclusion/exclusion criteria: would you also include trials with slightly different designs such as: stepped-wedge trials; adaptive trials?

**Author response 2. **Yes, trials of any design that meet the study definition of a late phase trial are eligible for inclusion; the additional examples you provided have now been included to the example list also. (New text in bold).

*late phase trials can include various trial designs (e.g. parallel, crossover, factorial, **adaptive, and stepped-wedge** designs)*

**Reviewer comment 3. **I suppose that SRs of trials would be excluded – although it would also be interesting to look if the use of COS is also addressed. Exclusion could be specifically mentioned.

**Author response 3. **There is on-going work examining uptake of COS in systematic reviews, and it is correct that systematic reviews are not eligible for inclusion in this review. This has been clarified in the methods section as follows:

***Systematic reviews and other study designs will also be excluded.***

**Reviewer comment 4. **Data extraction: if the use of a COS is not mentioned in the main paper, would it be safe to check the detailed protocol (in some areas, it might have been previously published in another journal) if no reference is made to an existing COS.

**Author response 4. **Data will now also be extracted from the trial protocol and/or registry on whether a COS was mentioned. This has ben added to the methods section as follows: ***Data will be extracted from the trial protocol or trial registry on whether a COS was mentioned.***

**Reviewer comment 5. **The search of existing COS is made in the COMET database. Is there any data to confirm that this is sufficient, i.e. that searching in parallel another database (eg PubMed) for published COS is not needed?

**Author response 5. **The COMET database is a repository of all completed, on-going, and planned COS. As such, we are confident that it is sufficient to search the COMET database only.

**Reviewer comment 6. **Questionnaire: how will you maximize response rate? Has the questionnaire been pilot-tested? Will there be close and open-ended questions?

**Author response 6. **To maximise survey response rates, survey invitation emails to trialists will be personalised and will be sent from the COMET Initiative  and the Medical Research Council (MRC) Trials Methodology Research Partnership, as these are recognised trial methodology initiatives and so may enhance trust in, and motivation to the complete, the survey. A reminder email will also be sent to all trialists after one week. This information has been included in the protocol as follows:

***To maximise survey response rate, survey invitation emails to trialists will be personalised and will be sent from the COMET Initiative and the Medical Research Council (MRC) Trials Methodology Research Partnership. A reminder email will also be sent to all trialists after one week.***

 The survey contains closed ended questions, with the exception of a final open-ended question asking trialists if there is anything else they wish to share. This has been clarified in the protocol :

***The survey includes closed ended questions asking about** trialists’ awareness of, and decisions to search for and use, a COS. **One open-ended question at the end of the survey asks participants if there is anything additional they wish to share**.*

**Reviewer comment 7. **The trialists’ views is essential to better understand the barriers and facilitators. Could there be any value of looking at some of the data extracted in the first part of the study, and identify from there factors associated with the (non)use of a COS? E.g. domain, type of funding, patient and public involvement…?

**Author response 7. **Thank you for this useful suggestion. For the purposes of this review, we have based the survey questions on preliminary data and information emerging from existing and on-going research regarding COS uptake. The findings of the current survey, incorporating both the review data and the survey, will provide a useful guide moving forward to examine and target barriers and facilitators to COS use.

# Peer review: Reviewer response for version 1

**DOI:** 10.21956/hrbopenres.14215.r27876

**Published:** 2020-09-30

**Authors:** Christine Bond

**Affiliations:** 1 Institute of Applied Health Sciences, School of Medicine, Medical Sciences & Nutrition, University of Aberdeen, Aberdeen, UK

This is an interesting well written protocol for a study to explore whether researchers are using core outcome sets and if not why not. The method involves identifying and critically reviewing recently published papers in major journals followed by a survey of authors. The paper explains why this is an important topic and the value of researchers using core outcomes, especially for evidence synthesis. I have just a few comments for the authors to consider.

 My main concern is about greater clarity of what this review will add to the existing studies which have shown poor uptake of COS in Cochrane, previous trials and recently funded HTA applications (do these HTA application need more explanation for international audiences?). This review is looking at major impact journals but why is that important or different. Also I agree the selected journals are subjectively important and influential but is there an objective justification for their selection? 

  Are the authors suggesting Journal Editors should use their influence to encourage use of COS, in the same way as reporting guidelines are required? 

 In many ways the triallists’ views are the most interesting aspect of the paper as a way of understanding what needs to be done in the future to promote use of COS. Building on that, could behavioural theory be used to inform the questions asked and allow identification of appropriate behaviour change interventions?

 Minor points

1. Is there a justification for the sample size of estimated 115 papers? This relates both to the generalisability of the findings and the value of the survey, especially for any sub sample analyses.
2. Is ‘pharmacologic trials’ a normal label? Often referred to more as investigational products or drug trials.
3. Under items to be extracted some e.g. disease name will not necessarily always be relevant.
4. For trials not reporting COS or part of COS is there a field for specifying the outcome that was actually used as well as any justification, for that decision?
5. First column page 4 has a longish paragraph on identifying if a relevant COS existed at the time of the trial. There is reference to time of designing the trial as being the base line. I am not sure how easy the date of the initial design would be to identify but one option might be to look at a relevant date of any grant application if the study had been externally funded.
6. In the same paragraph maybe add initials of the reviewer with prior experience and the second reviewer as presume they are members of the authorship team.
7. Will any inferential statistics be conducted on the survey results?
8. Who will be approached for an ethical opinion – presume it will be a University review board?
9. Who is funding this work?

Is the study design appropriate for the research question?

Yes

Is the rationale for, and objectives of, the study clearly described?

Partly

Are sufficient details of the methods provided to allow replication by others?

Yes

Are the datasets clearly presented in a useable and accessible format?

Not applicable

Reviewer Expertise:

Mixed methods health services researcher who has been part of a team developing a COS.

I confirm that I have read this submission and believe that I have an appropriate level of expertise to confirm that it is of an acceptable scientific standard, however I have significant reservations, as outlined above.

# Response

**Published:** 2020-12-09

**Authors:** Karen Matvienko-Sikar

**Affiliations:** University College Cork, Ireland

**Reviewer comment 1. **This is an interesting well written protocol for a study to explore whether researchers are using core outcome sets and if not why not. The method involves identifying and critically reviewing recently published papers in major journals followed by a survey of authors. The paper explains why this is an important topic and the value of researchers using core outcomes, especially for evidence synthesis. I have just a few comments for the authors to consider.

 My main concern is about greater clarity of what this review will add to the existing studies which have shown poor uptake of COS in Cochrane, previous trials and recently funded HTA applications (do these HTA application need more explanation for international audiences?). This review is looking at major impact journals but why is that important or different. Also I agree the selected journals are subjectively important and influential but is there an objective justification for their selection?

**Author response 1.**

 Thank you for your comments, the protocol has now been amended taking these into account.

 The rationale for examining the use of core outcome sets in major impact journals is that these journals are expected to publish trials of the highest quality, of clinical importance and relevance, and are likely to impact on policy and practice. This is different to Cochrane, which includes every trial, and HTA applications that may be more standardised. As such, this review will provide a different perspective on core outcome set use in trials, in terms of identifying uptake among those trials that are expected to be among those of the highest quality.

**Reviewer comment 2. **Are the authors suggesting Journal Editors should use their influence to encourage use of COS, in the same way as reporting guidelines are required?

**Author response 2. **The authors feel that Journal Editors are in a position to encourage and promote use of COS in trials. However, at protocol stage of this project we are not suggesting that Journal Editors use their influence to encourage COS because recommendations about how best to encourage COS use in trials will be derived from the data provided by the trialists in our survey.

**Reviewer comment 3. **In many ways the triallists’ views are the most interesting aspect of the paper as a way of understanding what needs to be done in the future to promote use of COS. Building on that, could behavioural theory be used to inform the questions asked and allow identification of appropriate behaviour change interventions?

**Author response 3. **We agree that the trialists survey responses will provide useful insights into how best to promote use of core outcome sets. Approaches such as the behaviour change wheel could be used to determine which behavioural determinants to target moving forward to enhance COS use.  This approach was used to inform an earlier qualitative study topic guide examining COS use, which has informed the survey used in the current study.

**Reviewer comment 4**

 Minor points

 Is there a justification for the sample size of estimated 115 papers? This relates both to the generalisability of the findings and the value of the survey, especially for any sub sample analyses.

**Author response 4.  **The sample size estimate was derived from initial scoping of the target journals, and is based on a pragmatic decision regarding the number of trials that are manageable to examine within the study timeframe but that are still representative of trials published by these journals. Sub sample analyses are not planned. The following has been added to the methods for clarity (new text in bold):

***Based on initial scoping of the target journals**, it is estimated that 115 trials, of various phases, have been published during in these journals during this timeframe and so this period has been chosen to ensure identification of a sufficient, yet pragmatically manageable number of recent trials for review by the review team.*

**Reviewer comment 5. **Is ‘pharmacologic trials’ a normal label? Often referred to more as investigational products or drug trials.

**Author response 5. **The manuscript has been amended to state pharmacologic/drug trials

**Reviewer comment 6.** Under items to be extracted some e.g. disease name will not necessarily always be relevant.

**Author response 6. **As the focus of the review is on clinical trials published in major medical journals, it is important that details including ‘disease name’ are extracted. However if during data extraction, where disease name is not applicable for a specific trial the reviewer will specify ‘not applicable’.

**Reviewer comment 7. **For trials not reporting COS or part of COS is there a field for specifying the outcome that was actually used as well as any justification, for that decision?

**Author Response 7. **The following is included in the ‘Data extraction and analysis’ section of the protocol to specify data extracted in instances where COS are not used: *For trials not reporting use of a COS, or reporting use of only some COS outcomes, the trial authors’ rationale and justification for the choice of outcomes used will be extracted from the published trial if reported.*

**Reviewer comment 8. **First column page 4 has a longish paragraph on identifying if a relevant COS existed at the time of the trial. There is reference to time of designing the trial as being the base line. I am not sure how easy the date of the initial design would be to identify but one option might be to look at a relevant date of any grant application if the study had been externally funded.

**Author Response 8. **Reference to identifying when the trial started is now removed and replaced with: ***Information on the date of registration of the trial and/or publication of the trial protocol will be identified, with the earliest date retained where there is more than one registration/protocol.***

**Reviewer comment 9. **In the same paragraph maybe add initials of the reviewer with prior experience and the second reviewer as presume they are members of the authorship team.

**Author Response 9. **We have now amended the process for COS identification, such that it will be conducted by pairs of reviewers who will work independently to identify COS in the COMET database and reach agreement by discussion. This has been updated below, though initials have not been included due to the on-going nature of this process.

***Checking the COMET database will be done independently by paired reviewers, with each pair identifying COS for a subset of trials. At least one member of each pair will have prior experience of identifying COS for specific populations and interventions. Agreement on identified COS will be reached by discussion between reviewer pairs and will be verified by the first and senior authors.***

**Reviewer comment 10. **Will any inferential statistics be conducted on the survey results?

**Author Response 10. **No the studies will be descriptively analysed to examine the numbers and percentage of trials using a COS, the numbers and percentage of trials that could have used a COS, and trialists’ awareness of, and decisions to search for and use, a COS.

**Reviewer comment 11. **Who will be approached for an ethical opinion – presume it will be a University review board?

**Author response 11. **Ethical approval has now been obtained from the University College Cork Social Research Ethics Committee. This information has now been added to the manuscript as follows: ***Ethical approval for this survey has been obtained from the University College Cork Social Research Ethics Committee (2020-137).***

**Reviewer comment 12. **Who is funding this work?

**Author response 12. **This work is not being directly funded. Karen Matvienko-Sikar is supported by a Health Research Board Applying Research into Policy and Practice Fellowship [HRB-ARPP-A-011]. Kerry Avery and Jane Blazeby are supported by the NIHR Biomedical Research Centre at University Hospitals Bristol and Weston NHS Foundation Trust and the University of Bristol. Funding support for Paula Williamson has now been included (Paula Williamson is supported by the Medical Research Council (MRC) Trials Methodology Research Partnership (grant reference MR/S014357/1). These funders are not directly funding this project however.
